# Frequency Effects with Visual Words and Syllables in a Dyslexic Reader

**DOI:** 10.1155/2005/427605

**Published:** 2006-01-09

**Authors:** Prisca Stenneken, Markus Conrad, Florian Hutzler, Mario Braun, Arthur M. Jacobs

**Affiliations:** Department of PsychologyFreie Universität BerlinGermany

**Keywords:** Acquired dyslexia, word frequency, syllable frequency, pseudohomophone effect, lexical decision, phonological processing of visual word forms

## Abstract

The present study investigated the nature of the inhibitory syllable frequency effect, recently reported for normal readers, in a German-speaking dyslexic patient. The reading impairment was characterized as a severe deficit in naming single letters or words in the presence of spared lexical processing of visual word forms. Three visual lexical decision experiments were conducted with the dyslexic patient, an unimpaired control person matched to the patient and a control group: Experiment 1 manipulated the frequency of words and word-initial syllables and demonstrated systematic effects of both factors in normal readers and in the dyslexic patient. The syllable frequency effect was replicated in a second experiment with a more strictly controlled stimulus set. Experiment 3 confirmed the patient’s deficit in activating phonological forms from written words by demonstrating that a pseudohomophone effect as observed in the unimpaired control participants was absent in the dyslexic patient.

## 1. Introduction

The aim of the present study was to investigate the nature of the syllable frequency effect in visual word processing of a German dyslexic patient. In normal readers, visual word processing performance as typically assessed in a lexical decision task is affected by lexical variables like word frequency. Here, decision latencies are shorter for high frequency as compared to low-frequency words. In addition, several case studies of impaired reading have reported preserved lexical effects, which are observed even in the presence of severely impaired oral reading (for an overview, see [7]). In contrast, effects of syllable frequency have been described only recently for the lexical decision performance of unimpaired readers [12]. Whereas high-frequency words elicit faster responses, high-frequency word-initial syllables lead to slower responses in lexical decision.

The inhibitory syllable frequency effect on lexical decision latencies has been established by Carreiras, Álvarez and de Vega [10] for normal readers in Spanish and has repeatedly been replicated (e.g. [35] for Spanish [31], for French). Comparable findings were reported for the German language that has a shallow orthography with rather regular grapheme phoneme correspondences [12]. Additional empirical evidence for the inhibitory syllable frequency effect came from studies in Spanish and German, demonstrating correlates of syllable frequency manipulations in event related brain potentials [6,21] and eye movements [22]. In sum, the findings characterize the inhibition in perceptual identification and lexical processing due to high-frequency word-initial syllables as a robust effect.

In their discussion of the origin of the inhibitory syllable frequency effect, Perea and Carreiras [35] suggested an important role played by the target word's higher frequency syllabic neighbors (words that share the initial syllable with the target and that are of higher

[1] This research was partly supported by a grant from the German Research Foundation (DFG) to A.M.J. (Ja 823/3-1).
*Corresponding author: Prisca Stenneken, Ph.D., Department of Psychology, Freie Universität Berlin, Habelschwerdter Allee 45, 14195 Berlin, Germany. Tel.: +49 30 858 55622; Fax: +49 30 858 55620; E-mail: PStenn@zedat.fu-berlin.de.

ISSN 0953-4180/05/$17.00 © 2005 – IOS Press and the authors. All rights reserved word frequency than the target). Accordingly, the authors explained the observed inhibition as related to lexical access referring to an interactive activation model of visual word recognition [32]: Once such a model would be implemented with a level of syllabic representation, the representations of all syllabic neighbors would be activated via the processing of the initial syllable of the target word. All activated lexical entries would be competing candidates. Thus, the syllable neighbors would interfere with the processing of the target word by a mechanism of lateral inhibition at the level of whole word entries. Targets with high-frequency word-initial syllables should activate a larger number of lexical candidates; the increased competition among these would make the identification of the target word more difficult. Up to now, the most often cited computational models of visual word recognition do not comprise syllabic representations and are therefore challenged by empirical effects of syllabic information on word processing [11,18,25,44,48].

In contrast, recent word naming experiments in Spanish showed that the effect of initial syllable frequency seems to change its direction when overt production is required: Word and non-word naming was faster when their initial syllable was of high frequency [9,35]. Perea and Carreiras [35] explained the facilitation effect by assuming a switch of the locus of effect to motor output in the naming task. When speech output is concerned, high frequency syllables being well learned units of speech would be accessed and produced more rapidly. Thus, at least in Spanish the inhibitory effect of syllable frequency that is influential during lexical access would not generalize to performance in the naming task. Taken together, investigating normal reading there is a growing body of evidence that syllables are functional units in both word production and visual word recognition.

Recently, patient studies have emerged investigating effects of syllable frequency in single word processing in aphasic patients with a phonological encoding impairment [28,43,40] or in patients with apraxia of speech [1]. Whereas most studies with unimpaired readers investigated syllable frequency effects in lexical decision tasks, the patient studies focused on tasks requiring phonological output like picture naming, word repetition and word reading. Because there are only few reports of manipulating syllable frequency in patient case studies, we will summarize the main findings here.

Wilshire and Nespoulous [43] investigated the influence of word-final syllable frequency on the phonological errors of two French-speaking aphasic individuals with a phonological encoding impairment. Results showed that syllable frequency did not affect the error rates in word reading and repetition tasks. Moreover, the aphasic errors did not show a preference for more frequent syllables compared to targets. These findings contradict the facilitatory syllable frequency effect observed in unimpaired readers. According to the authors, the results provide no evidence for an individual representation and access of syllables in phonological output preparation.

In contrast, a subgroup of the aphasic patients with a phonological encoding impairment investigated by Laganaro [28] showed a syllable frequency effect in a variety of tasks (picture naming, word reading and word repetition). The errors in three (out of seven) Italian- speaking or Spanish-speaking patients showed a tendency towards more frequent syllables replacing a less frequent syllable in the target. Moreover, these patients were more impaired in repeating non-words composed of low-frequency syllables compared to those with high-frequency syllables. An additional analysis of substitution errors in a French speaking aphasic patient, revealed a tendency for more frequent syllables in the aphasic errors as compared to the targets. These results support the idea of syllable representations.

Similarly, Aichert and Ziegler [1] reported systematic influences of syllable frequency in a patient study. They investigated 10 German-speaking patients with apraxia of speech in a word repetition task. Results showed a significant effect of syllable frequency on error rates: lower error rates were obtained for items with extremely high syllable frequencies. However, error rates quickly increased with decreasing syllable frequencies. With regard to the underlying deficit of apraxia of speech, the authors conclude that the patients fail to retrieve correctly the syllabic motor patterns. This is in line with impairment at the phonetic encoding level, which is assumed to include a mental store of motor programs for high-frequency syllables.

In sum, the above studies dealing with syllable frequency provide evidence for syllables as functional units in phonological encoding and output preparation. An open question is whether syllabic units play a functional role in visual word processing irrespective of accessing the phonological word form. In written words, a syllable can be considered both an orthographic and a phonological unit. Note that both in Spanish and in German, the two languages that were used in most of the studies focusing on effects of syllable frequency in visual word recognition, one orthographic syllable has almost always only one phonological correspondent and vice versa, because both are shallow orthographies. Therefore, the reported studies do not allow any conclusions about whether documented effects of syllable frequency are due to phonological or orthographic processing. Very recently, this question has been addressed by Álvarez and colleagues [2] who reported effects of priming with phonological syllabic neighbors in lexical decision, suggesting that phonological syllables are involved in the process of silent reading and that the observed syllable frequency effects have a strong phonological component.

To further explore the nature of this inhibitory syllable frequency effect, we studied a German speaking dyslexic patient (US) who shows a deficit in generating the phonological forms of visually presented letters and words. We knew from pilot investigations of this patient, that lexical effects were preserved in visual lexical decision indicating a preserved mechanism for lexical access. In contrast, oral reading was severely impaired. Even reading of short words was characterized by a letter-by-letter reading procedure with frequent misnaming of single letters. The pattern of impairments suggests that the phonological word form is not accessed by the time the lexical decision is made. The present study explored whether the inhibitory syllable frequency effect is observed irrespective of this patient's impaired access to phonology in visual word processing. If the inhibitory syllable frequency effect on word processing latencies is linked to activation of output phonological representations from visual forms, then it should be diminished in the patient. By contrast, a preserved syllable frequency effect would provide support for a role of syllabic units in visual word processing independently of full access of the phonological word form.

This question was addressed in a series of lexical decision experiments, all performed by the dyslexic patient, a control participant matched to the patient and a group of unimpaired control participants. Experiment 1 manipulated syllable frequency and word frequency orthogonally; Experiment 2 further examined the effect of syllable frequency, using a more tightly controlled set of materials. A third experiment was conducted to further determine the patient's deficit in a decision task, comparable to that in Experiments 1 and 2. Here, the stimulus set of non-words contained pseudohomophones. Given the patient's deficit in accessing phonology from print, our prediction was that she should not show the pseudohomophone effect that is typically observed in normal readers.

## 2. Case study

The patient US, a 70-year-old woman, is right-handed and native German speaker; before the onset of the disease, she had worked as a medical doctor. She was tested in visual word processing experiments 5.8 years post onset of a CVA. CT scanning in the acute phase of the illness revealed an infarction of the left posterior cerebral artery including the temporal branches of the postero-lateral thalamus, and of the right posterior cerebellar artery.

The initial neurological examination revealed a severe reading disorder, a moderate agraphia, mild anomia, right hemianopia, and impaired short-term memory function. A moderate sensorimotor deficit was diagnosed for the right arm and leg. Standardized testing with the Aachen Aphasia Battery [20] at one month post-onset revealed no clear evidence for an aphasic disorder: No or minimal impairment was observed in the Token Test, in word and sentence repetition, and in auditory comprehension. The subtests of written language processing showed a severe impairment. In the acute stage, picture naming was moderately impaired. Here, isolated naming failures occurred in picture naming or in describing drawings of every day situations. In object naming, the patient reported a tip-of-the-tongue state. She produced adequate circumlocutions (e.g., for the picture of a squirrel: "it's an animal that lives on the trees and eats nuts") or showed conduite d'approche phenomena (producing the correct response with repetition).

At the time of the experimental testing, only a mild anomia persisted in spontaneous speech production. This was observed only rarely, especially with very low-frequency words and proper names. The agraphic symptoms had diminished within the first two months. Sensorimotor function was improved; the patient showed only a light unstableness. The hemianopia was compensated up to 25–30$^\circ$ to the right hemispace. As the most prominent symptom remained a severe reading disorder. In reading aloud, the patient employed a letter-by-letter reading strategy; the time taken to read words aloud was extremely long and increased with the number of letters. In naming single letters, the patient made frequent and inconsistent errors. She produced self-corrections and was sometimes able to access segmental information only via the whole word form. These aspects are illustrated below in examples of the patient's word reading:

Stimulus 1: BLUME [flower]
US: "the first one is a B, that's easy. But the second letter.... this one is always so hard to remember. It's always the same.... and then, U, then N, and then E. UN, UNE. no, it's an M. UME and B.... oh, it's BLUME. it's an L"
Stimulus 2: LAGER [store]
US: "L, then A, LA... I have no idea.... LAG... LAGER"

When presented with German compounds consisting of three or more nouns, the patient was not able to reliably read the word form but rather guessed; reading sentences was hardly possible. A more detailed characterization of the reading impairment is given in the following section on the patient's baseline assessment.

### *2.1. Baseline assessment of the reading disorder*

**Letter Identification.** Two tasks were administered investigating the processing of single letters, a categorization and a naming task. The categorization task required the grouping of single written letters according to their identity (e.g. different case and font realizations like A, a, A, **a**) without naming the letters. The patient was able to correctly group letters of different font types and in upper and lower case according to their identity (accuracy 40 out of 40 letters). In contrast, oral naming of written letters (either letter names or the corresponding sounds were scored as correct) was severely impaired; 9 out of 26 letters were correctly named (when excluding self-correction and multiple guessing responses). In sum, the findings suggest that the patient's deficit does not concern the early identification of the visual letter shapes, but rather accessing the names (or respective phonemes) of individual letters.

**Rhyme decision.** In this task, 40 word pairs consisting of 80 monosyllabic words with three to six letters were presented simultaneously in upper case letters on a computer screen. In half of the word pairs, the two stimuli had an identical phonological rhyme, in the other half the two words did not rhyme. In all pairs, the orthographic forms of the two words were different and the amount of orthographic overlap between the two words was matched for the rhyme pairs and the non-rhyming stimulus pairs (e.g., rhyme pair: BERG – WERK; non-rhyme pair: BERG – WERT). The stimuli were presented on the computer screen until a response was given; however, the instructions emphasized strongly that the participant should not read the words aloud and rather try to decide as fast as possible by key presses whether the two displayed word forms rhymed or not, irrespective of the word's spelling. Accuracy was 27 (out of 40) word pairs; this finding suggests an impaired access to the phonological form of written words.

**Phonological Decision.** This task required US to indicate whether a written stimulus sounded like a legal German word or not. The stimulus set consisted of 20 words, 20 pseudohomophones (requiring a yes-response) and 20 pseudowords (requiring a no-response). The words were monosyllabic and disyllabic German nouns, three to seven letters long, and had a mean frequency of 32.55 per million [5]. Pseudohomophones were derived from the words by the exchange of vowels (e.g., SÄULE -> SEULE) or by the exchange of consonants (e.g., HAND -> HANT) without altering the phonological form. Pseudowords were derived from pseudohomophones by the exchange of vowels (e.g., HAND -> HONT). The stimuli were presented in lower case letters on a computer screen (with capitalization as appropriate in German). Accuracy of the dyslexic patient was 16 out of 20 (80% correct) for words, 7 out of 20 (35% correct) for pseudohomophones and 14 out of 20 (70% correct) for pseudowords. Because this task is not contained in the typical set of baseline tests, we refer to the performance of an unimpaired control group [23]. Unimpaired participants performed this task correctly for words (100% correct), and with a high rate of correct responses for pseudohomophones (96% correct) and pseudowords (91% correct). Thus, in comparison to the unimpaired control participants, the dyslexic patient showed a generally lower performance, with an especially high rate of erroneous rejections (misses) for the pseudohomophones. This suggests that the patient does not sufficiently activate the phonological information of visual word forms in this decision task.

**Word, non-word and sentence repetition.** No impairment was observed for the patient in repeating auditorally presented words and non-words (accuracy 30 out of 30). In sentence repetition, accuracy was 19 out of 20; the incorrect repetition of one sentence may be attributed to the slightly reduced memory span. Accordingly, a general phonological processing deficit including auditory material could be excluded as the source of the patient's phonological difficulties.

**Phoneme synthesis.** This task investigated whether the patient was impaired in producing word forms

Table 1
Performance (number of correct responses out of total, percentage in brackets) in the baseline assessment tasks of the patient US (chance level was 50% over all items in the rhyme decision and the phonological decision tasks)

| Task | Correct Responses |
|---|---|
| letter identification | 40/40 (100) in categorization |
| | 9/26 (34.6) in naming |
| rhyme decision | 27/40 (67.5) |
| phonological decision | 16/20 (80) words |
| | 7/20 (35) pseudohomophones |
| | 14/20 (70) pseudowords |
| repetition | 30/30 (100) words |
| | 19/20 (95) non-words |
| phoneme synthesis | 18/20 (90) |

based on single phonemes. It required the composition of whole words from auditorally presented single phonemes. The individual phonemes were presented by the investigator in a regular pace (about one phoneme per second), e.g. /t/, /a/, /b/, /l/, /e/ from which the patient had to produce the full word form *table*. Half of the 20 stimuli had a regular phonological-orthographic structure and half were irregular (i.e. contained phonemes corresponding to more than one letter). Stimuli were presented with increasing length, ranging from two to seven phonemes. The patient correctly reported 18 (out of 20) words; one error occurred with a regular and one with an irregular item of seven phonemes. These errors may be related to reduced short-term memory capacity not suggesting a specifically impaired phoneme synthesis.

In sum, the baseline assessment suggests that the deficit of this dyslexic patient can be isolated to impaired access of the phonological form of written words or letters (see summary of findings in Table 1). The patient's performance was severely impaired in tasks combining written stimulus material with phonological processing, like rhyme and phonological decision and letter naming. Of particular note, the patient's impaired letter naming suggests a connection between the reading impairment and the anomia that persisted for proper names and very low-frequency words. A more general output phonological deficit, however, was ruled out by the patient's good performance in word and non-word repetition and phoneme synthesis tasks. In general, phonological processing was spared, as long as no written stimulus material was involved.

Whereas naming latencies for words depended on the number of letters, no such effect of word length on latencies or error rates was observed in the decision tasks (rhyme decision and phonological decision). Moreover, residual processing of visual material at the segmental level was indicated by the results of the letter categorization task, which required written material but no phonological output.

The deficit displayed by patient US suggests that she processes letters serially when reading aloud. Considering the increase of errors and time needed with number of letters, US's deficit can be characterized as letter-by-letter reading (see [7] for a review). It has been suggested that letter-by-letter-readers have difficulty in establishing the abstract identity of visually presented letters [4,13]. In contrast, the pattern of deficits for US (especially the good performance in the letter categorization task) suggests that she can successfully access representations of abstract letter identity. A similar case of acquired dyslexia was patient MS described by Mycroft and colleagues [33] who showed a spared processing of the abstract identity of letters in the presence of severely impaired naming of visual letters and words, comparable to that of US in the acute stage. Those authors suggest that, in contrast to most other letter-by-letter readers, MS was suffering from a disconnection between preserved abstract identity representations of letters and the representation of their names in the output lexicon. Even though the performance of US in naming letters and words is somewhat better than in MS, her testing suggests a comparable underlying deficit in accessing phonological forms from print.

## 3. Experiment 1: Effects of word frequency and syllable frequency

The empirical basis for the first experiment is the inhibitory effect of syllable frequency observed in word processing for unimpaired readers [12,35]. Results showed that high-frequency word-initial syllables slow down the perceptual identification and lexical decision performance. The aim of the present experiment was, first, to demonstrate spared lexical processing the dyslexic patient US, which should be indicated by a facilitatory effect of word frequency on decision latencies. Moreover, we wanted to further explore the nature of this inhibitory syllable frequency effect in a patient with a severe impairment in activating the phonological forms of visually presented words. Therefore, patient US was investigated using a lexical decision experiment orthogonally manipulating word frequency and word-initial syllable frequency and her performance was compared with a matched control person and a control group.

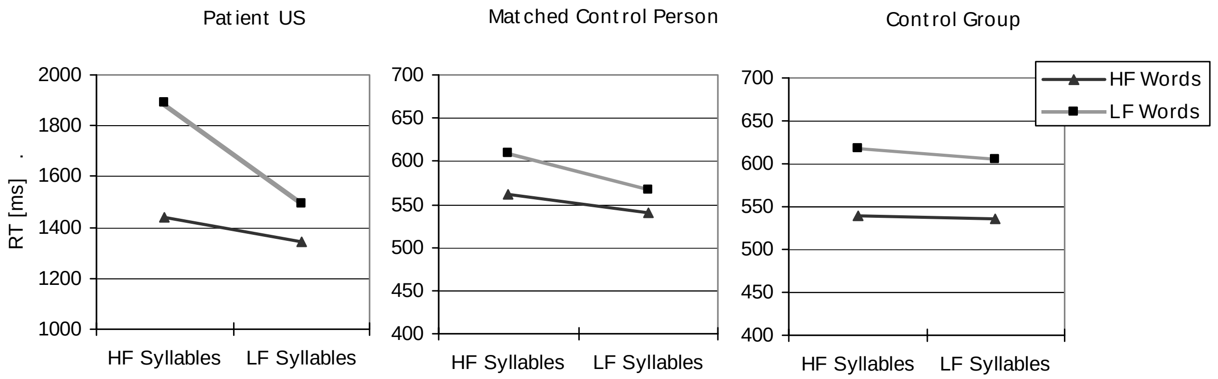

Fig. 1. Facilitatory effects of word frequency and inhibitory effect of word-initial syllable frequency on lexical decision latencies for the patient US (left panel), the matched control person (middle panel), and the control group (right panel).

### *3.1. Method*

**Participants**

Participants were the patient US (for details, see Case Study), a group of student-age unimpaired readers and an unimpaired control participant, matched to the patient in age, gender, handedness and education. The matched control participant was a 71-year-old right-handed native speaker of German with university level education. The student-age group comprised 24 persons (14 female, 10 male), aged between 20 and 28 years (average 23.6). They were students from Eichstätt-Ingolstadt University and received credits for course requirements for their participation. All participants were native speakers of German, right handed by self-report and had normal or corrected to normal vision.

**Stimulus Material**

The stimulus set consisted of 200 items, subdivided into 100 bi-syllabic content words. Word frequency and syllable frequency were orthogonally manipulated. The two sub-groups of word stimuli were controlled for length (five or six letters), word class (nouns, verbs, adjectives), number of orthographic neighbors, number of high-frequency orthographic neighbors, bigram frequency and frequency of the second syllable. In addition, 100 non-words served as filler items; these were legal pseudowords derived from the word stimuli by substituting one or two letters (for stimulus characteristics, see Appendix A).

**Procedure**

Participants were asked to perform a lexical decision task on a set of visually presented words and non-words. They indicated whether the displayed stimulus was a legal German word or not by a two-choice response (button press). Each trial started with a fixation cross (visible for 500 ms) followed by the stimulus presented in lower case letters in black font in the center of the computer screen (17" ProNitron 17/200 Monitor) with white background color. The stimuli remained visible until the response occurred. Participants were asked not to read the stimuli aloud and were encouraged in case of doubt to make a fast guessing response. They were instructed to respond as fast as possible without making errors.

### *3.2. Results and discussion*

Data analyses were based on reaction times for correct responses; extreme reaction time values (defined as less than or greater than two standard deviations from the average) were excluded from analyses.

The dyslexic patient performed the visual lexical decision task with an accuracy of 79% for words and 77% for non-words. Reaction time analyses showed effects of word frequency and syllable frequency (see Fig. 1, left panel).[2] The overall analysis showed a significant effect for word frequency $F(1,75) = 4.76$, $p < 0.05$, the factor syllable frequency showed a trend $F(1,75) = 3.27$, $p = 0.074$, and the interaction was not significant ($p = 0.281$). Thus, results showed a facilitatory influence of word frequency with faster responses to high-frequency words (1387 ms [z-score $-0.23$]; standard error (se) 85) as compared to low-frequency words (1722 ms [z-score 0.29]; se 114). Here, also fewer errors occurred for high-frequency

[2] A first analysis of the patient data has been presented at the Science of Aphasia Conference [41].

words (6 out of 50) compared to low-frequency words (15 out of 50). In contrast, responses to words beginning with a high-frequency syllable were slower (1667 ms [z-score 0.21]; se 117) as compared to words beginning with low-frequency syllables (1400 ms [z-score $-0.21$]; se 74). Here, no consistent numerical effects were observed in error patterns.

The matched control person performed this task with an accuracy of 97% for words (all errors occurred with low-frequency words beginning with low-frequency syllables) and 98% for non-words. Response latencies (Fig. 1, middle panel) showed significant main effects for word frequency $F(1, 96) = 5.92$, $p < 0.05$ and syllable frequency $F(1, 96) = 4.61$, $p < 0.05$, the interaction was not significant ($p = 0.499$). As expected, the effect of word frequency was facilitatory with shorter reaction times for high-frequency words (551 ms [z-score $-0.24$]; se 10.00) as compared to low-frequency words (589 ms [z-score 0.25]; se 11.90). The frequency of the initial syllable had an inhibitory effect, with longer reaction times for words beginning with high-frequency syllables (586 ms [z-score 0.21]; se 12.73) as compared to low-frequency syllables (552 ms [z-score $-0.22$]; se 8.69).

For the student control participants, the accuracy of lexical decision was 95% for words and 96% for non-words, with accuracy for high-frequency as compared to low-frequency words of 98% vs. 92%, and with comparable accuracy for items with low-frequency and high-frequency first syllables (95% each). Reaction times of correct responses were submitted to an analysis of variance; results (see Fig. 1, right panel) revealed a facilitatory effect of word frequency $F(1, 23) = 52.33$, $p < 0.001$ with faster responses to high- frequency words (537 ms [z-score 0.40]; se 18.67) as compared to low-frequency words (612 ms [z-score $-0.40$]; se 19.17). Syllable frequency had an inhibitory effect $F(1, 23) = 4.84$, $p < 0.05$, indicated by slower responses to high-frequency word-initial syllables (578 ms [z-score $-0.40$]; se 19.50) as compared to low-frequency syllables (571 ms [z-score 0.40]; se 18.33). The interaction between the two factors was not significant ($p = 0.242$).

Summarizing the results of the dyslexic patient, both the facilitatory effect of word frequency and the tendency of an inhibitory syllable frequency effect were in line with the findings described in the literature of unimpaired participants. Moreover, the results of the younger and the age-matched control participants replicated the syllable frequency effect described before for the German language [12]. Given the specific impairment for the dyslexic patient, the present findings seem to suggest that syllable frequency influences visual word processing independently of having overt access to the phonological form.

The patient's deficit in accessing the phonological form of written words had been determined in baseline testing, indicated by the very low performance in letter naming, rhyme decision, and phonological decision. Moreover, baseline testing revealed no impairment in repeating auditorally presented words and non-words, so that a phonological deficit at peripheral stages in speech production was excluded as the source of the patient's phonological difficulties.

To obtain additional evidence specifying the phonological access deficit in US, an oral reading task was performed by the patient on a subset of stimuli used in the lexical decision task. The comparison of the two tasks should specify the time course of accessing the phonological word forms in the patient. Stimulus presentation was computer-assisted like that in the lexical decision task with unlimited presentation times. Results showed that in contrast to the ease with which lexical decision is performed, reading aloud of single words is extremely difficult for the patient. When considering all responses without time limit, reading accuracy (without considering self-corrections) in the patient was 56% for words, 41% for non-words. The patient tried to identify single letters and to combine these to produce the phonological form in an extremely slow letter-by-letter reading procedure. This is reflected in mean naming latencies; the time to finish the first syllable was on average 3946 ms (and significantly increased compared to lexical decision latencies $t(209) = 16.20$, $p < 0.001$) so that response latencies cannot reliably be interpreted. Most importantly, the finding of low accuracy and slow responses in the oral reading of the same stimuli that gave rise to the lexical and syllable frequency effects suggest that the phonological word form is not fully accessed by the time the lexical decision is made.

## 4. Experiment 2: Replicating the syllable frequency effect

A second lexical decision experiment was administered to the dyslexic patient, the matched control person and a control group. The aim of this experiment was to replicate the syllable frequency effect identified in the dyslexic patient using a more strictly controlled stimulus set. In contrast to Experiment 1, word fre-

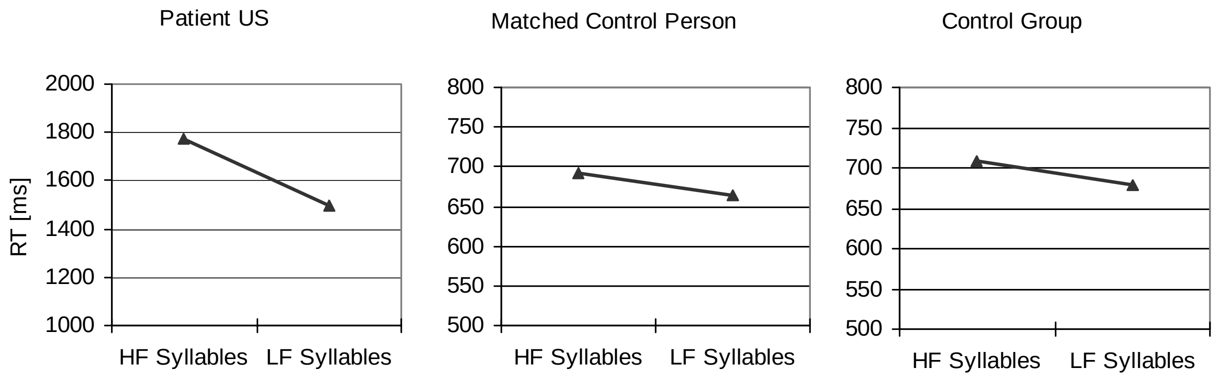

Fig. 2. Inhibitory effects of word-initial syllable frequency on lexical decision latencies for the patient US (left panel), the matched control person (middle panel), and the control group (right panel).

quency was kept constant in this experiment allowing control for additional features of the stimulus set, like length of the first syllable, word class and construction of non-word stimuli. A replication of the syllable frequency effect with the patient with this stimulus set should provide additional evidence for a functional role of syllables in visual word processing.

### 4.1. Method

Participants were the patient US and the matched control person in Experiment 1. The student-age control group consisted of 18 unimpaired readers (6 female, 11 male, reported in [21]) with an age range of 20 to 44 years (average 28). All other characteristics of the control participants were as described for Experiment 1. Experimental procedure and data analysis were comparable to that of Experiment 1.

The stimulus set contained 110 bi-syllabic nouns (55 with high-frequency, 55 with low-frequency word-initial syllables) and 110 non-words. High frequency and low frequency syllable words were selected as described for Experiment 1. In contrast to Experiment 1, word frequency was held constant and only nouns were used. Non-words were created by randomly combining existing first syllables with existing second syllables of German words. The resulting letter strings had to be orthographically legal and pronounceable (for stimulus characteristics, see Appendix B).

### 4.2. Results and discussion

Results from patient US confirmed the inhibitory effect of word-initial syllable frequency on lexical decision responses (Fig. 2, left panel). Analyses of correct responses showed linger reaction times for words beginning with high-frequency syllables (1776 ms, [z-score 0.21]; se 114) than for words beginning with low-frequency syllables (1497 ms, [z-score $-0.21$]; se 81) with $t(85) = 2.00$, $p < 0.05$. Accuracy was 78% for words with high-frequency first syllables and 80% for words with low-frequency first syllables.

The matched control person performed the task with accuracy of 96% for high-frequency syllables and 93% for low-frequency syllables. Reaction time analyses confirmed slower responses for words beginning with high-frequency syllables (692 ms [z-score 0.21]; se 9.79) than for words beginning with low-frequency syllables (664 ms [z-score 0.21]; se 8.04) with $t(102) = 2.19, p < 0.05$ (Fig. 2, middle panel).

Similarly, the results of the control group, in which the overall accuracy for words was above 98%, showed a clear syllable frequency effect (Fig. 2, right panel); responses were slower to words with high-frequency first syllables (709 ms [z-score = 0.20]; se 21) than to words with high-frequency first syllables (679 ms [z-score = $-0.20$]; se 17; $t(17) = 3.86, p < 0.001$).

These results complement the findings from Experiment 1. Again, a syllable frequency effect was observed in the dyslexic patient and in the unimpaired control participants irrespective of age. Based on the evidence from Experiment 1, the more strictly controlled stimulus set in Experiment 2 allowed us to investigate the syllable frequency effect in words of one frequency class and the word class of nouns. In addition, syllable length was controlled, so that high frequency syllables and low frequency syllables were comparable concerning amount of segmental information. Thus, systematic effects of syllable length or structure on lexical decision latencies (for a discussion, see [16,39]) were excluded in the newly controlled stimulus set. Together with previous studies reporting a syllable frequency effect in visual word processing (in Spanish, French and Italian), the present replications for German and the fact that the effect is observed in the patient allow for characterizing the effect as robust and well established.

## 5. Experiment 3: Pseudohomophone effect

Experiments 1 and 2 established a systematic effect of syllable frequency on visual lexical decision latencies in US, observed in the presence of a severely impaired access to phonological form representations of written words. Given these findings, we wanted to test our assumption about the patient's phonological processing deficit for written word forms in an experiment that was methodologically as similar as possible to the two preceding experiments. The patient's language profile in the baseline assessment had confirmed this specific phonological impairment. However, these results relate the deficit to overt phonological output generation. To examine a possible deficit in a task requiring silent reading, i.e., generation of the phonological form from written words without overt production, we constructed an additional experiment in which (like in Experiments 1 and 2) the stimulus material includes an inherent manipulation of the relevant factor.

For comparison with the two previous experiments, Experiment 3 required a lexical decision. The critical manipulation was that non-word stimuli contained a subset of pseudohomophones (these were phonologically identical to German words, but were non-words according to orthographic structure). If US was impaired in activating the phonological form during visual word recognition, then there should be a reduced or no effect of pseudohomophones on decision latencies in contrast to the control group.

The pseudohomophone effect was devised to investigate word recognition in English by Rubenstein and co-workers [37]. They presented pseudohomophones (e.g., FEAL) and non-words as spelling-controls that did not sound like words (e.g., FEEP). The pseudohomophone FEAL is phonologically identical to the English word FEEL, but its spelling is different. The spelling-control FEEP is neither phonologically nor orthographically identical to the real word FEEL. Results showed that decision latencies in correct rejections of non-words were larger for pseudohomophones than for spelling-controls. Thus, the pseudohomophone effect denotes a phonological interference effect, arising even in tasks that theoretically can be performed without phonological processing [24,25]. This characterization of the effect as due to automatic phonological activation from written word forms has been confirmed in a number of studies [8,15,38,46,47]. In the present experiment, we expected to observe the pseudohomophone effect for the control group, whereas in the patient's performance no reliable difference should be observed in responses to pseudohomophones and spelling controls.

### 5.1. Method

**Participants**

The patient US, the matched control person (as in Experiments 1 and 2), and a group of unimpaired control participants performed this lexical decision task. The control group of unimpaired participants comprised 12 persons (8 female, 4 male), aged between 19 and 27 years (average 22). Other characteristics of the control participants are as described for Experiment 1 and 2.

**Procedure**

Participants were asked to perform a lexical decision task on a set of visual words and non-words. Stimuli were presented in upper case letters in black font in the center of a computer screen (Formac Pro Nitron 17") with white background color. Every trial started with a 500 ms fixation mark in the center of the screen, followed by the target stimulus for 200 ms. Participants indicated by pressing a response key, whether the displayed letter string was a legal German word or not. They were instructed not to read the stimuli aloud and were encouraged to make a fast guessing response if uncertain. They were instructed to respond as fast as possible without making errors.

**Stimulus Material**

The stimulus set contained 160 stimuli (80 words and 80 non-words). Out of 80 word stimuli, 40 served as fillers and accordingly were excluded from all subsequent analyses. Non-word stimuli comprised 40 pseudohomophones and 40 spelling-controls. All four subgroups (filler words, base words, pseudohomophones, spelling-controls) were equally distributed into stimuli of four and five letters in length.

Pseudohomophones and spelling-controls were constructed according to the criteria proposed by Martin [30], i.e., the two categories were controlled for frequency, number of neighbors, word length and number of syllables. Pseudohomophones and spelling-controls were derived from the same base word by changing (i.e., replacing, adding, or removing) one letter in the

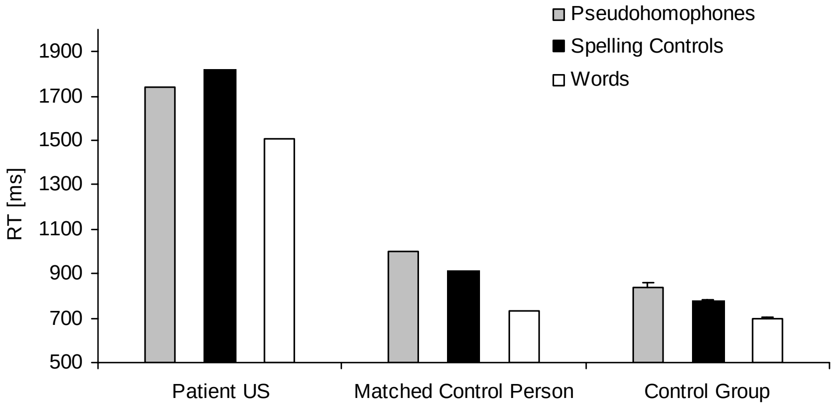

Fig. 3. Homophony and lexicality effects in the dyslexic patient US (left panel), in the matched control person (mid panel), and in the control group (right panel).

same position. Pseudohomophones thus differed from the respective base words in spelling, but not in phonological form, whereas spelling-controls differed from base words in both phonology and spelling. Moreover, pseudohomophones and spelling-controls were matched for scores in standard orthographic similarity indices [27] and for neighborhood size, neighborhood frequency, higher frequency neighbors, frequency of higher frequency neighbors, number of letters, number of syllables, bigram neighbors, bigram frequency, and bigram count (for stimulus characteristics, see Appendix C).

### 5.2. Results and discussion

Data analyses focused on reaction times of correct responses with extreme reaction time values (defined as less than or greater than two standard deviations from the average) excluded. US performed the task with 68% accuracy; the rate of correct responses was 67% for pseudohomophones and 62% for spelling controls. In reaction time analyses, the effect of stimulus category, i.e. pseudohomophones, spelling controls and words, showed a trend $F(1, 71) = 2.54$, $p = 0.086$. Most important, pairwise comparisons revealed no significant difference between pseudohomophones (1743 ms [z-score 0.10]; se 84) and spelling controls (1819 ms [z-score 0.25]; se 131) with $t(48) = 0.49$, $p = 0.626$ (cf. Fig. 3, left panel). The lack of a pseudohomophone effect for US indicates that in non-words, the phonological similarity to words did not affect the rejection latencies in lexical decision. Additional comparisons of word stimuli (1505 ms [z-score $-0.36$]; se 88) to the two categories of non-words showed marginally significant effects of the item's lexical status; shorter reaction times are observed for words as compared to pseudohomophones ($t(49) = 1.95$, $p = 0.056$) and spelling controls ($t(45) = 2.01, p = 0.051$).

Accuracy in the matched control person was 93%; rate of correct responses for the pseudohomophones was 88% and for the spelling controls was 95%. Latencies of correct responses (see Fig. 3, middle panel) showed an effect of stimulus category, i.e. pseudohomophones, spelling-controls and words $F(2, 109) = 27.39$, $p < 0.001$. A clear pseudohomophone effect was observed $t(70) = 2.14$, $p < 0.05$, with average reaction times for pseudohomophones of 1001 ms [z-score 0.65]; (se 27.31) and for spelling-controls of 910 ms [z-score 0.18]; (se 25.83). In addition, results showed a lexicality effect: responses to words (733 ms [z-score $-0.73$]; se 25.18) were significantly shorter compared to pseudohomophones $t(72) = 6.65$, $p < 0.001$ and spelling controls $t(72) = 6.07, p < 0.001$.

The group of unimpaired control participants performed this task with 94% accuracy; the rate of correct responses was 95% to words, 90%, to pseudohomophones and 97% for spelling controls. Reaction time analyses of correct responses revealed a significant effect of stimulus category (see Fig. 3, right panel), i.e. pseudohomophones, spelling-controls and words $F(2, 22) = 42.17, p < 0.001$. Pairwise comparisons between categories revealed a pseudohomophone effect $t(11) = 4.31$, $p < 0.05$, with longer response latencies to pseudohomophones (842 ms [z-score = 0.65]; se 56) as compared to spelling-controls (768 ms [z-score = $-0.04$]; se 46). Moreover, lexicality had an effect on response latencies; these were shorter for words (693 ms [z-score = $-0.63$]; se 40) as compared to pseudohomophones $t(11) = 7.53$, $p < 0.001$ and spelling controls ($t(11) = 7.42, p < 0.001$.

As expected, the results for the unimpaired control person and the control group replicated the pseudohomophone effect for German non-words. Moreover, this experiment established the phonological interference effect for a stimulus set constructed under strong stimulus selection criteria [27,30]. Results are in line with the assumption of an automatic phonological recoding in normal visual word processing even in tasks that do not require phonological output [42,45].

In contrast, the dyslexic patient showed comparable decision latencies for pseudohomophones and spelling-controls. This finding is compatible with an impairment of activating phonological information from visual words. Moreover, results suggest that this impairment does not only affect output phonology but also implicit phonological processing of visual words. The null effect can be reliably interpreted, because the overall performance is well above chance level and the observed lexicality effect indicates successful lexical access. Accordingly, the absence of a pseudohomophone effect in the dyslexic patient suggests that the she has not accessed the phonological form at the time of the lexical decision, at least not to an extent causing interference with ongoing lexical processing.

## 6. General discussion

The present study examined the inhibitory syllable frequency effect observed in lexical decision of dyslexic and unimpaired participants. More specifically, we addressed whether this effect would also be observed when the access of the phonological representations of written words is severely impaired. This was studied in US a dyslexic patient whose deficit could be attributed in accessing the phonological forms for written words and letters. The results of Experiment 1 and 2 established an inhibitory syllable frequency effect on lexical decision latencies in the dyslexic patient. These findings strongly suggest that the inhibition due to processing of words with high-frequency syllables does not necessarily depend on activation of the phonological word form.

Our assertion is based on the specific locus of the dyslexic patient's impairment. This was observed in a series of tests, indicating that (a) the deficit was specific to written material and did not affect phonological processing for auditorally presented stimuli (cf., spared phoneme synthesis and word/non-word repetition); (b) it did not relate to a generally impaired processing of letters (cf., spared letter categorization) suggesting that abstract letter identities could be established; (c) it affected overt reading (impaired oral reading and letter naming) and also 'silent reading' without generation of phonological output (cf., impaired rhyme decision and phonological decision). For this last point, additional evidence was obtained in analyses of Experiment 1 and 3. Experiment 1 showed that, in contrast to the ease with which lexical decision is performed, reading aloud of single words is extremely difficult for the patient (Exp. 1). Given the increased response latencies for oral reading, we assumed that the phonological form is not fully accessed at the time the lexical decision is made. Experiment 3 confirmed the impairment in a word processing task that does not require phonological output by demonstrating the absence of a pseudohomophone interference effects in the patient's lexical decision latencies.

In contrast to impaired phonological activation, patient US showed spared lexical processing in all three experiments. Her lexical decision performance was above chance level and lexical factors had significant effects on her decision latencies. In sum, the impairment for US is characterized by spared lexical processing in the presence of impaired phonological access from visual word forms. This can be related to a larger group of patient studies (overview in Ellis [14]; Patterson & Marcel [34]) indicating that written words can be processed to some extent in the presence of impaired phonological mediation (see also the case study of Rapp and colleagues [36], reporting an analogous deficit in language production: their patient is impaired in oral naming of objects although writing the names correctly). Most importantly, the pattern of deficits in patient US suggests that she can successfully access letter identities to reliably activate lexical representations for visually presented words (see also patient MS in [33]).

Given this patient's deficit, we need to answer the question of why an inhibitory syllable frequency effect is observed. A first possible explanation of the syllable frequency is that it as a purely phonological effect. The observed inhibition could be attributed to phonological interference of syllable neighbors with the target word. As illustrated above, it is, however, unlikely that phonological forms are sufficiently activated for US. Her poor oral reading (Experiment 1) and, more importantly, the lack of a pseudohomophone interference on word recognition (Experiment 3) provide evidence that the phonological form is not available in visual word (or letter) processing at the time the lexical decision was made.

Alternatively, one could argue that the syllable frequency effect is caused by rudimentary activation of phonological forms. It could be the case that some phonological information is activated in the patient even though the complete phonological word form is not accessible. However, this assumption raises the problem of why this activation would be strong enough to produce a syllable frequency effect but not sufficient to cause phonological interference (as indicated by the absence of the pseudohomophone effect in the patient).

According to a third interpretation, the syllable frequency effect could reflect inhibition at a lexical level, caused by early visual, orthographic processing of syllabic units. This assumption would be in line with spared lexical processing in the patient as indicated by above chance level lexical decisions, and spared effects of lexicality and word frequency. Both observations indicate intact lexical processing of visual words. Further evidence for this account comes from studies of unimpaired readers. As mentioned in the Introduction, the syllable frequency effect in visual lexical decision has been related to the concept of orthographic neighborhood [35]. Perea and Carreiras conducted a post hoc analysis of the inhibitory syllable frequency effect in their lexical decision experiment. Results pointed to a systematic effect of the number of higher frequency syllabic neighbors (words that share a syllable with the target being of higher word frequency than the target). The authors therefore attributed the inhibitory effect observed in the processing of words beginning with high-frequency syllables to lexical interference. Interestingly, analyses did not reveal a similar influence of syllabic neighbors in a naming task. The faster responses to words with high-frequency syllables observed here seem to reflect a phonological facilitation process. The inhibitory effect of the target word's orthographic neighborhood on word recognition and processing latencies has also been confirmed in several studies that did not directly manipulate syllable frequency [3,17,35].

The assumption of lexical interference can be demonstrated in the framework of successful computational models of the recognition of monosyllabic words (e.g. multiple read-out model, MROM [18,26]; dual-route-cascaded model, DRC [11]). As pointed out before, these models are not implemented with a level of syllabic representations. By analogy to the influence of orthographic neighbors, one could however assume a comparable model with an architecture including a layer of syllabic representations in which a word with a high-frequency initial syllable would activate a larger number of *syllabic* neighbors. Thus, for words beginning with high-frequency syllables there would be a larger or a more rapid increase in overall lexical activation.

Applying these considerations to the present results of patient US seems plausible. The patient's word reading, proceeding in a letter-by-letter fashion with delayed and error-prone naming of single letters, suggests a disruption of phonological representations below the word level. The disruption does not necessarily include syllabic units; rather, the experiments in the present study point to the relevance of syllabic units in visual word processing. Additional support for this view is provided by a case study by Lesch and Martin [29]. Their dyslexic patient ML showed a good performance on tasks involving syllabic units as compared to impaired processing of smaller units (e.g. onset, body) or single segments. The authors attributed the reading impairment to a disruption of representations below the level of the syllable. Thus, in general, the characterization of US's deficit would be compatible with assuming the inhibitory syllable frequency effect to reflect lexical competition caused by the activation of syllabic neighbor representations.

In sum, the present findings indicate that syllables may serve as functional units in visual word recognition, causing inhibition at the lexical processing level. The results of the dyslexic patient showed that effects of syllable frequency could be observed in spite of impaired grapheme to phoneme conversion or activating phonology from visual word forms.

## Acknowledgements

We express our gratitude to US for her willingness to participate in this study. We thank Brenda Rapp and two anonymous reviewers for helpful comments on a former version of the manuscript.

## Appendix

Appendix A. Stimulus characteristics in Experiment 1

| | HF word / HF syllable | HF word / LF syllable | LF word / HF syllable | LF word / LF syllable |
|---|---|---|---|---|
| First syllable-F (per million) | 12567 | 360 | 10493 | 92 |
| Length of first syllable (letters) | 2.24 | 3.04 | 2.24 | 2.80 |
| Second syllable-F (per million) | 2088 | 2624 | 2000 | 2254 |
| Word frequency | 210.49 | 220.95 | 4.57 | 4.73 |
| Neighborhood size (N) | 1.60 | 1.52 | 1.72 | 1.84 |
| Higher frequent neighbors (N) | 0.12 | 0.12 | 0.76 | 0.72 |
| Bigram frequency (per million) | 16785.6 | 20637.6 | 92224.3 | 13035.5 |

HF: high-frequency. LF: low-frequency. Syllable-F: Syllable-frequency.

Appendix B. Stimulus characteristics in Experiment 2 (cited from [21])

| | HF syllable | LF syllable |
|---|---|---|
| First syllable-F (per million) | 7051 | 132 |
| Length of first syllable (letters) | 2.65 | 2.78 |
| Second syllable-F (per million) | 12907 | 15148 |
| Word frequency (per million) | 9.05 | 8.51 |
| Word length (letters) | 5.44 | 5.42 |
| Neighborhood size (N) | 2.85 | 2.80 |
| Higher frequent neighbors (N) | 1.29 | 1.36 |
| Bigram frequency (per million) | 9655 | 8746 |

HF: high-frequency. LF: low-frequency. Syllable-F: Syllable-frequency.

Appendix C. Stimulus characteristics in Experiment 3

| | Pseudo-homophones | Spelling controls |
|---|---|---|
| Word frequency (per million) | 3.94 | 3.94 |
| Word length (syllables) | 1.33 | 1.33 |
| Word length (letters) | 4.5 | 4.5 |
| Neighborhood size (N) | 2.30 | 2.92 |
| Frequency neighbors (FN) | 204.85 | 148.98 |
| Higher frequency neighbors (HFN) | 1.23 | 1.40 |
| Bigram count (BiC) | 48.5 | 52.65 |
| Bigram frequency (BiF) | 2983.97 | 3829.53 |
| Bigram neighbors (BN) | 9.08 | 11.85 |
